# Zinc Pyrithione Improves the Antibacterial Activity of Silver Sulfadiazine Ointment

**DOI:** 10.1128/mSphere.00194-16

**Published:** 2016-09-14

**Authors:** Catlyn Blanchard, Lauren Brooks, Katherine Ebsworth-Mojica, Louis Didione, Benjamin Wucher, Stephen Dewhurst, Damian Krysan, Paul M. Dunman, Rachel A. F. Wozniak

**Affiliations:** a Department of Microbiology and Immunology, University of Rochester School of Medicine and Dentistry, Rochester, New York, USA; b Department of Pediatrics, University of Rochester School of Medicine and Dentistry, Rochester, New York, USA; c Department of Ophthalmology, University of Rochester School of Medicine and Dentistry, Rochester, New York, USA; University of Nebraska Medical Center

**Keywords:** *Acinetobacter baumannii*, *Pseudomonas aeruginosa*, *Staphylococcus aureus*, biofilm, silver sulfadiazine, wound, zinc pyrithione

## Abstract

*Pseudomonas aeruginosa*, *Acinetobacter baumannii*, and *Staphylococcus aureus* are commonly associated with biofilm-associated wound infections that are recalcitrant to conventional antibiotics. As an initial means to identify agents that may have a greater propensity to improve clearance of wound-associated bacterial pathogens, we screened a Food and Drug Administration-approved drug library for members that display bactericidal activity toward 72-h-established *P. aeruginosa* biofilms using an adenylate kinase reporter assay for bacterial cell death. A total of 34 compounds displayed antibiofilm activity. Among these, zinc pyrithione was also shown to reduce levels of *A. baumannii* and *S. aureus* biofilm-associated bacteria and exhibited an additive effect in combination with silver sulfadiazine, a leading topical therapeutic for wound site infections. The improved antimicrobial activity of zinc pyrithione and silver sulfadiazine was maintained in an ointment formulation and led to improved clearance of *P. aeruginosa*, *A. baumannii*, and *S. aureus* in a murine model of wound infection. Taken together, these results suggest that topical zinc pyrithione and silver sulfadiazine combination formulations may mitigate wound-associated bacterial infections and disease progression.

**IMPORTANCE** Topical antimicrobial ointments ostensibly mitigate bacterial wound disease and reliance on systemic antibiotics. Yet studies have called into question the therapeutic benefits of several traditional topical antibacterials, accentuating the need for improved next-generation antimicrobial ointments. Yet the development of such agents consisting of a new chemical entity is a time-consuming and expensive proposition. Considering that drug combinations are a mainstay therapeutic strategy for the treatment of other therapeutic indications, one alternative approach is to improve the performance of conventional antimicrobial ointments by the addition of a well-characterized and FDA-approved agent. Here we report data that indicate that the antimicrobial properties of silver sulfadiazine ointments can be significantly improved by the addition of the antifungal zinc pyrithione, suggesting that such combinations may provide an improved therapeutic option for the topical treatment of wound infections.

## Abstract (precis)

Topical antimicrobial ointments ostensibly mitigate bacterial wound disease and reliance on systemic antibiotics. Yet studies have called into question the therapeutic benefits of several traditional topical antibacterials, accentuating the need for improved next-generation antimicrobial ointments. Yet the development of such agents consisting of a new chemical entity is a time-consuming and expensive proposition. Considering that drug combinations are a mainstay therapeutic strategy for the treatment of other therapeutic indications, one alternative approach is to improve the performance of conventional antimicrobial ointments by the addition of a well-characterized and FDA-approved agent. Here we report data that indicate that the antimicrobial properties of silver sulfadiazine ointments can be significantly improved by the addition of the antifungal zinc pyrithione, suggesting that such combinations may provide an improved therapeutic option for the topical treatment of wound infections.

## INTRODUCTION

Wound sites frequently serve as foci for bacterial colonization and invasive disease, particularly among postsurgical, burn, and diabetic patient populations. Indeed, surgical site wound infections are reported to occur in approximately 11% of patients undergoing major elective surgery, resulting in a 5-fold increase in hospital remittance and a 2-fold to 3-fold increase in mortality (1). Similarly, burn site infections increase patient mortality by 5%, whereas diabetic wound infections are associated with increased risk of limb amputation and a 12.7% increase in mortality rates (2–4). The development of novel bacterial decolonization strategies has the potential to limit the incidence of wound infection and corresponding rates of morbidity and mortality.

The ESKAPE (*Enterococcus faecium*, *Staphylococcus aureus*, *Klebsiella pneumoniae*, *Acinetobacter baumannii*, *Pseudomonas aeruginosa*, and *Enterobacter* species) pathogens are among the most common causative agents of acute and chronic wound infections (5, 6). Collectively, these organisms account for approximately 57% of burn wound infections (7, 8). Moreover, *P. aeruginosa* or *S. aureus* or both are also the predominant causes of surgical wound site infections among patients undergoing spine (9), coronary artery bypass grafting (10), abdominal (11), and orthopedic (12) procedures. Their propensity to form biofilms, a bacterial physiological growth state that is recalcitrant to conventional antibiotics and host defense processes, has been well documented to contribute to chronic diabetic ulcer pathogenesis and may also play a role in mediating infection in burn and postsurgical patients (13).

Wound site infection treatment is often further complicated by compromised host vascular perfusion that limits systemic antibiotic drug delivery and jeopardizes efficacy, thus accentuating the importance of effective topical antimicrobials. In that regard, silver-based topical antimicrobials are commonly used as an adjunct to systemic antibiotics for the prevention and treatment of wound infections (reviewed in reference 14). Silver ions nonspecifically react with bacterial proteins and DNA, thereby leading to multiple mechanisms of antimicrobial action (15, 16). Such promiscuity accounts for silver’s broad-spectrum antimicrobial activity toward both Gram-positive and -negative bacterial species and is thought to contribute to its antibiofilm activity. One of the most common preparations of medicinal silver, silver sulfadiazine, is the active component of numerous wound care-associated products, including ointments, creams, and bandages as well as coatings on indwelling catheters, representing a $237 million dollar industry in the United States (17–19). Despite such widespread use, numerous clinical studies have called into question the impact of silver sulfadiazine treatment. Although silver sulfadiazine shows improved antimicrobial activity in comparison to placebo, the agent displays no statistically significant improvement with respect to the prevention of infection in comparison to nonsilver comparators such as collagenase ointment, hydrocolloid dressings, and bee honey (20–22). Such reports, combined with rising concerns over the emergence of silver resistance, have prompted interest in developing improved topical therapeutic options (23).

With that goal in mind, we screened a Food and Drug Administration (FDA)-approved drug library for agents with bactericidal activity toward established *P. aeruginosa* biofilm-associated cells. The antifungal, zinc pyrithione (ZnPT), was active against *P. aeruginosa*, but its antimicrobial potency was less than that of silver sulfadiazine (SSD). Conversely, ZnPT’s antimicrobial activity against *S. aureus*, *A. baumannii*, and six other bacterial pathogens tested was superior to that of SSD, suggesting that ZnPT-SSD combinations would exhibit broader-spectrum antimicrobial activity than either agent alone. Interestingly, the combination of ZnPT and SSD exhibited additive antimicrobial effects on planktonic and biofilm-associated *S. aureus*, *P. aeruginosa*, and *A. baumannii*. Likewise, the combination of ZnPT plus SSD in a topical formulation displayed significantly improved antimicrobial activity toward each bacterial species in a murine model of acute wound infection. Considering that both ZnPT and SSD are already active antimicrobial components of numerous topical formulations, their combination represents a novel and potentially more effective treatment alternative for wound infections.

## RESULTS

### Identification of zinc pyrithione as an antibiofilm agent.

A library of 853 FDA-approved drugs was screened for agents that displayed antimicrobial activity toward 72-h-established *P. aeruginosa* biofilms using an adenylate kinase (AK)-based bactericidal assay (24). Screening results revealed that a total of 34 library members exhibited a ≥2-fold increase in AK signal in comparison to mock-treated biofilms, indicating that they elicited a bactericidal response to biofilm-associated *P. aeruginosa* cells (Table 1). Among these were 28 antibiotics belonging to eight distinct classes, four antituberculosis agents, and two nonantibiotics that have been previously developed for other therapeutic indications. Ten of the identified antibiotics, tobramycin, doripenem, aztreonam, ciprofloxacin, besifloxacin, ofloxacin, norfloxacin, lomefloxacin, moxifloxacin, and levofloxacin, are known antipseudomonal agents with current clinical use in the United States. Eight additional fluoroquinolones, two quinolones, three macrolides, tigecycline, spectinomycin, streptomycin, three tetracyclines, four antituberculosis agents, and two nonantibiotics, bleomycin sulfate and zinc pyrithione, were also identified, suggesting that they may be valuable *P. aeruginosa* antibiofilm agents.

**TABLE 1:** Selleck library screening hits

Drug class	Drug	Avg fold change in AK signal^a^
Aminocyclitol	Spectinomycin hydrochloride	6.6 (± 3.5)
		
Aminoglycoside	Streptomycin sulfate	44.0 (± 0.7)
	Tobramycin	50.0 (± 10.4)
		
β-Lactam	Doripenem hydrate	56.0 (± 1.4)
	Aztreonam	12.0 (± 9.9)
		
Fluoroquinolone	Ciprofloxacin	29.0 (± 0.7)
	Clinafloxacin	36.0 (± 24)
	Balofloxacin	32.0 (± 0.2)
	Besifloxacin HCl	34.0 (± 12)
	Danofloxacin mesylate	46.0 (± 15)
	Enrofloxacin	41.0 (± 17)
	Levofloxacin	38.0 (± 2.8)
	Lomefloxacin hydrochloride	59.0 (± 23)
	Moxifloxacin hydrochloride	43.0 (± 5.7)
	Nadifloxacin	35.0 (± 20)
	Norfloxacin	56.0 (± 6.3)
	Ofloxacin	39.0 (± 9.8)
	Sitafloxacin hydrate	41.0 (± 0.6)
	Sparfloxacin	40.0 (± 0.7)
		
Quinolone	Pefloxacin mesylate	41.0 (± 1.3)
	Sarafloxacin HCl	27.0 (± 6.9)
		
Glycylcycline	Tigecycline	38.0 (± 23)
		
Macrolide	Azithromycin	50.0 (± 1.9)
	Clarithromycin	59.0 (± 30)
	Erythromycin	63.0 (± 5.1)
		
Tetracycline	Oxytetracycline	6.6 (± 2.1)
	Methacycline hydrochloride	62.0 (± 20)
	Tetracycline HCl	56.0 (± 14)
		
Antitubercular	Rifabutin	33.0 (± 5.4)
	Rifampin	16.0 (± 9.8)
	Rifapentine	4.5 (± 1.6)
	Rifaximin	20.0 (± 20)
		
Nonantibiotic	Bleomycin sulfate (anticancer)	3.1 (± 0.5)
	Zinc pyrithione (antifungal)	24.0 (± 11)

a Data represent average fold increases in adenylate kinase (AK) release in comparison to mock treatment results (standard deviations are indicated in parentheses).

As an initial means to validate our screening results, seven compounds that were readily available (ciprofloxacin, spectinomycin, tobramycin, rifampin, levofloxacin, tetracycline, and zinc pyrithione) were retested for antimicrobial activity toward established *P. aeruginosa* biofilms. To do so, biofilms were formed, challenged with increasing concentrations of each agent (0 to 256 µg·ml^−1^), and then plated for enumeration of the remaining viable biofilm-associated bacteria. Results revealed that all of the compounds tested did indeed result in a ≥0.37 log_10_ reduction in the level of biofilm-associated cells at the initial screening concentration (50 µM; 16 to 32 µg·ml^−1^, depending on the agent tested) and exhibited a dose-dependent decrease in biofilm cell viability at the higher concentrations tested, confirming that our initial screen performed as expected (Table 2). Differences in antibiofilm potency were also observed, suggesting that some subsets of antibiotics are likely to outperform others in the treatment of *P. aeruginosa* biofilm-associated infections. Ciprofloxacin and levofloxacin exhibited the most potent antimicrobial activity toward biofilm-associated cells at virtually all concentrations tested, resulting in 1.8- to 4.6-log and 1.1- to 6.8-log decreases in levels of biofilm cells, respectively. Tobramycin and zinc pyrithione produced more-modest 0.7- to 2.4-log and 0.3- to 2.2-log reductions in levels of biofilm-associated cells. The antibiotics spectinomycin, rifampin, and tetracycline produced only marginal decreases in levels of biofilm cells, yielding maximum reductions of 0.75- to 1.5-log in levels of biofilm-associated cells at the highest concentrations tested (164 to 256 µg·ml^−1^).

**TABLE 2:** Validation of screening hits

Drug	No. of biofilm-associated cells (log_10_) at drug concn (µg·ml^−1^) of:							
	0	4	8	16	32	64	128	256
Ciprofloxacin	8.67 (± 0.20)	6.81 (± 0.20)	5.94 (± 0.80)	6.19 (± 0.90)	5.05 (± 1.20)	4.13 (± 0.20)	4.27 (± 1.90)	4.03 (± 0.20)
Levofloxacin	9.26 (± 0.10)	8.14 (± 0.20)	6.03 (± 0.30)	5.91 (± 0.90)	5.74 (± 0.50)	3.57 (± 0.20)	5.12 (± 1.90)	2.41 (± 0.60)
Tobramycin	8.52 (± 0.00)	7.77 (± 0.10)	7.46 (± 0.02)	6.85 (± 0.80)	5.86 (± 0.30)	6.18 (± 0.30)	6.28 (± 0.80)	6.09 (± 1.50)
Zinc pyrithione	8.78 (± 0.10)	8.46 (± 0.02)	7.70 (± 0.10)	7.32 (± 0.60)	7.66 (± 0.01)	6.05 (± 0.70)	6.80 (± 0.10)	6.61 (± 0.10)
Rifampin	8.46 (± 0.30)	8.97 (± 0.20)	8.46 (± 0.30)	8.67 (± 0.20)	8.09 (± 0.10)	8.15 (± 0.90)	7.62 (± 0.30)	7.70 (± 0.10)
Spectinomycin	8.46 (± 0.30)	8.17 (± 0.01)	7.60 (± 0.10)	7.94 (± 0.10)	7.51 (± 0.01)	7.64 (± 0.10)	7.49 (± 0.02)	7.51 (± 0.01)
Tetracycline	8.95 (± 0.20)	8.52 (± 0.40)	8.46 (± 0.30)	8.57 (± 0.50)	8.07 (± 0.20)	7.78 (± 0.20)	7.87 (± 0.10)	7.36 (± 0.10)

Considering that one of our overarching goals was to identify novel therapeutics for the treatment of wound-associated bacterial infections, we considered that zinc pyrithione (ZnPT) may be a viable topical candidate to explore further because (i) ZnPT is currently a successful topical treatment of seborrheic dermatitis with a favorable safety profile (25), (ii) bacterial ZnPT resistance is slow to develop and unlikely to confer cross-resistance to systemic antibiotics (26), and (iii) ZnPT has been previously shown to have excellent antibiofilm activity toward *A. baumannii*, another common wound infection-associated organism (27).

### Spectrum of activity of zinc pyrithione (ZnPT).

As an initial means to evaluate the potential of ZnPT-based wound infection intervention approaches, we measured the agent’s spectrum of antimicrobial activity against other bacterial species commonly associated with wound infections, including *Escherichia coli* and each of the ESKAPE pathogens. As shown in Table 3, standard MIC testing revealed that ZnPT demonstrated antimicrobial activity toward all species evaluated. The agent exhibited the most potent activity (MICs between 1 and 2 µg·ml^−1^) toward *E. coli*, *S. aureus*, *Klebsiella pneumoniae*, *A. baumannii*, *Enterococcus faecium*, and *E. faecalis*, whereas it exhibited less antimicrobial activity toward both *Enterobacter cloacae* (MIC, 4 µg·ml^−1^) and *P. aeruginosa* (16 µg·ml^−1^). Moreover, with the exception of *P. aeruginosa*, ZnPT exhibited between 2- and 16-fold-increased antimicrobial activity in comparison to silver sulfadazine (SSD). Given that SSD exhibited improved activity against *P. aeruginosa*, whereas ZnPT displayed increased activity toward the other bacterial species tested, we considered that the combination of zinc pyrithione and silver sulfadiazine may demonstrate a broader-spectrum antimicrobial effect than either compound in isolation.

**TABLE 3:** MIC testing

Bacterial species^a^	MIC (µg·ml^−1^)	
	Zinc pyrithione	Silver sulfadiazine
*Escherichia coli* (8307)	1	16
*Staphylococcus aureus* (UAMS-1)	1	16
*Klebsiella pneumoniae* (CKP4)	2	16
*Acinetobacter baumannii* (98-37-09)	2	4
*Pseudomonas aeruginosa* (PAO1)	16	8
*Enterococcus faecium* (824-05)	2	8
*Enterococcus faecalis* (V583)	2	8
*Enterobacter cloacae* (clinical isolate)	4	16

a Strains are indicated in parentheses.

### Zinc pyrithione demonstrates additive activity with silver sulfadiazine toward *P. aeruginosa*, *S. aureus*, and *A. baumannii*.

As a first test of the antimicrobial effects of ZnPT and SSD in combination, fractional inhibitory concentration (FIC) testing was performed in a checkerboard format using planktonic *P. aeruginosa*, *S. aureus*, and *A. baumannii* to evaluate whether the combination displayed equivalent, antagonistic, or improved effects on three organisms frequently associated with wound infections. Results revealed additive antimicrobial effects on *P. aeruginosa* (FIC = 0.55 ± 0.02), *S. aureus* (FIC = 0.52 ± 0.02), and *A. baumannii* (FIC = 0.66 ± 0.13), suggesting that the combination of ZnPT and SSD may be more effective than either agent alone. Next, we evaluated whether the additive effects of ZnPT and SSD in combination extended to biofilm-associated *P. aeruginosa*, *A. baumannii*, and/or *S. aureus*. To do so, biofilms were established for each organism and then treated with increasing concentrations of each agent alone or in combination (0 to 128 µg·ml^−1^ of the total active ingredient[s]), and the corresponding biofilm-associated cell viability was quantified for each treatment condition.

**FIG 1:**
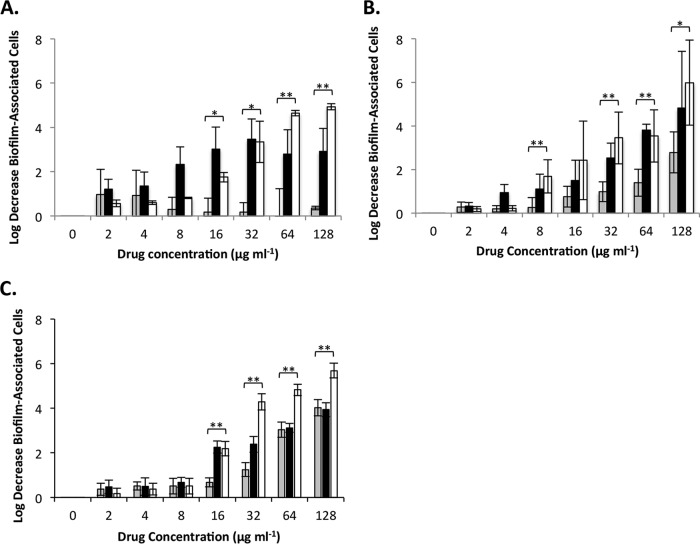
Antibiofilm effects of zinc pyrithione and silver sulfadiazine individually and in combination against *S. aureus*, *A. baumannii*, and *P. aeruginosa*. The log decrease in the number of biofilm-associated cells following treatment with 2-fold-increasing concentrations (0 to 128 µg·ml^−1^) of silver sulfadiazine (gray bars), zinc pyrithione (black bars), or silver sulfadiazine-zinc pyrithione combination (white bars) in comparison to mock-treated (DMSO) biofilms for *S. aureus* (A), *A. baumannii* (B), and *P. aeruginosa* (C). Error bars represent standard deviations. Significant differences between the results seen with silver sulfadiazine and silver sulfadiazine-zinc pyrithione are indicated. *, *P* ≤ 0.05; **, *P* ≤ 0.01 (Student’s *t* test).

As shown in Fig. 1A, *S. aureus* biofilms treated with the combination of ZnPT and SSD displayed a greater reduction in biofilm-associated cell numbers than biofilms treated with an equivalent amount of either agent alone at concentrations of 64 and 128 µg·ml^−1^. More specifically, SSD (alone) exhibited a consistent and yet modest 0.25 to 1 log reduction of biofilm-associated cell numbers at virtually all concentrations tested (2 to 128 µg·ml^−1^). ZnPT (alone) demonstrated a dose-dependent increase in antibiofilm activity, which showed a maximum 3.5-log reduction in biofilm-associated cell numbers at 32 µg·ml^−1^ but no further decrease at higher concentrations tested. However, the combination of ZnPT and SSD demonstrated a consistent dose-dependent increase in antimicrobial activity at concentrations above 4 µg·ml^−1^ (2 µg·ml^−1^ ZnPT and 2 µg·ml^−1^ SSD), resulting in a maximum of 5 log reduction of biofilm-associated *S. aureus* numbers at 128 µg·ml^−1^ (64 µg·ml^−1^ of each agent).

Similar results were observed for *A. baumannii*-treated biofilms (Fig. 1B). SSD (alone) demonstrated a modest dose-dependent decrease in *A. baumannii* biofilm-associated cell numbers, with a maximum of 2.8-log reduction at 128 µg·ml^−1^. ZnPT (alone) also demonstrated a dose-dependent increase in antimicrobial activity, which reached a maximum of an approximately 4.8 log reduction at 128 µg·ml^−1^. The combination of ZnPT and SSD exhibited more potent activity toward *A. baumannii* biofilms at 8 µg·ml^−1^ than either agent alone, and this improvement in antimicrobial activity continued in a dose-dependent manner, resulting in a 6 log reduction in cell numbers at the highest concentration tested (128 µg·ml^−1^ [64 µg·ml^−1^ ZnPT and 64 µg·ml^−1^ SSD]).

Likewise, measures of effects of ZnPT and/or SSD treatment on *P. aeruginosa* biofilms revealed that the combination exhibited increased antibiofilm activity in comparison to either compound alone (Fig. 1C). Indeed, both SSD treatment (alone) and ZnPT treatment (alone) demonstrated a dose-dependent reduction in biofilm-associated cells, reaching a maximum of a 4-log decrease in biofilm-associated cell numbers at 128 µg·ml^−1^. The combination exhibited a dose-dependent increase in activity in comparison to either agent alone beginning at 32 µg·ml^−1^, reaching a maximum 5.7 log reduction at 128 µg·ml^−1^ (64 µg·ml^−1^ ZnPT and 64 µg·ml^−1^ SSD).

Taken together, these results indicate that the combination of ZnPT and SSD exhibited increased antimicrobial activity toward *P. aeruginosa*, *S. aureus*, and *A. baumannii* in both the planktonic and biofilm growth states in comparison to either agent alone. Thus, we considered that the topical application of ZnPT-SSD in an ointment preparation may have therapeutic value.

### Ointment zone-of-inhibition measures.

To evaluate whether ZnPT and SSD were compatible in ointment format, both agents were formulated alone and in combination in standard polyethylene glycol (PEG)-based vehicle and assessed for activity in antimicrobial plate assays, as previously described (28). To perform the assessment, *P. aeruginosa*, *S. aureus*, or *A. baumannii* cells were spread on agar plates, PEG-based ointment containing 1% dimethyl sulfoxide (DMSO) (vehicle) or 1% SSD or 0.25% ZnPT or the SSD-ZnPT combination (1% SSD–0.25% ZnPT) was applied to the center of the plate, and the resulting zone of growth inhibition was measured following overnight incubation. It should be noted that 1% SSD was selected for these studies to reflect the concentration commonly provided in commercially available ointment preparations, whereas 0.25% ZnPT was selected for these studies because higher concentrations of the agent appeared insoluble in PEG ointment (data not shown).

**FIG 2:**
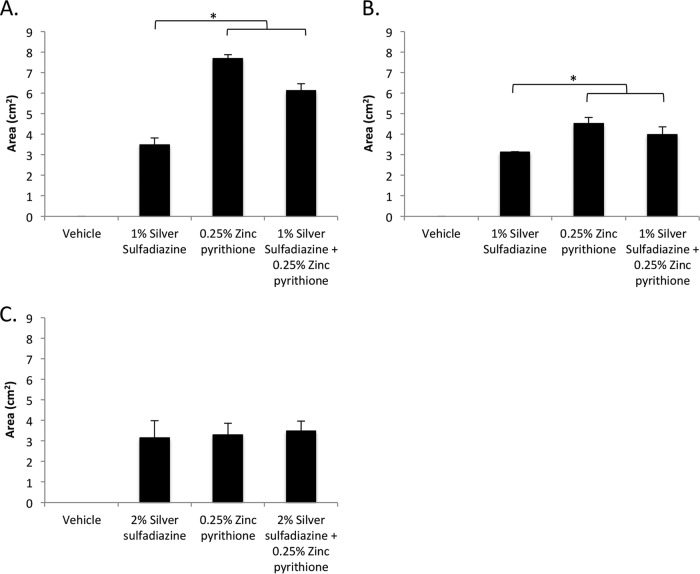
Antimicrobial zone of inhibition. The average zones of inhibition (*y* axis [measured in square centimeters]) of PEG-based ointments containing the indicated agent or mixture (*x* axis) toward *S. aureus* (A), *A. baumannii* (B), and *P. aeruginosa* (C) are plotted. Significant differences in the sizes of the inhibition zones in comparison to the results seen with 1% or 2% silver sulfadiazine are indicated. *, *P* ≤ 0.05 (Student’s *t* test).

Measures of each treatment’s zone of inhibition revealed that while vehicle alone did not affect the growth of any species tested, SSD and ZnPT, applied both alone and in combination, exhibited similar antimicrobial properties, suggesting that the formulation did not antagonize, or diminished only slightly, the activity of either agent. More specifically, against *S. aureus*, 1% SSD generated a 3.49 (± 0.33)-cm^2^ zone of inhibition, whereas 0.25% ZnPT generated an average zone of inhibition of 7.68 (± 0.19) cm^2^. The combination of 1% SSD and 0.25% ZnPT displayed a 6.12 (± 0.35)-cm^2^ zone of inhibition, indicating a slight reduction in the activity of the combination in comparison to ZnPT alone (Fig. 2A). Similarly, tests of *A. baumannii* revealed that 1% SSD and 0.25% ZnPT elicited zones of inhibition of 3.12 (± 0.02) cm^2^ and 4.51 cm^2^ (± 0.30), respectively. The combination of 1% SSD and 0.25% ZnPT produced a similar zone of inhibition of 3.98 cm^2^ (± 0.38) (Fig. 2B). Surprisingly, the use of 1% silver sulfadiazine did not reproducibly result in a measurable zone of inhibition for *P. aeruginosa* strain PAO1 cells (data not shown). As a consequence, the concentration was increased to 2% SSD to achieve a measurable zone of clearance of 3.16 (± 0.82) cm^2^, which was similar to the zone sizes seen with 0.25% ZnPT (3.30 cm^2^ [± 0.55]) and ZnPT and SSD in combination (3.48 cm^2^ [± 0.48]) (Fig. 2C). Taken together, these data indicate that ZnPT and SSD in combination were compatible in the PEG-based ointment formulations tested here. Nonetheless, in comparison to planktonic suspension testing results, the effects of the combination in an ointment formulation did not appear additive (i.e., SSD–ZnPT did not produce an increased zone of growth inhibition in comparison to either agent alone). Such a disparity could reflect limitations in the release characteristics of the two agents in ointment formulation or indicate that the physicochemical properties of the coexistence of the two agents in ointment formulation somehow negate the additive properties observed earlier in liquid format. To distinguish between these two possibilities, at least in part, the antimicrobial activity of ointment compilations was tested in a murine dermal wound model and the results were compared.

### Silver sulfadiazine and zinc pyrithione combination-based ointment demonstrated effective antimicrobial activity *in vivo*.

A murine acute dermal wound model was used to evaluate the topical antimicrobial properties of PEG-based ointments containing vehicle or SSD or ZnPT or the combination of SSD plus ZnPT. To do so, dorsal wounds were created and inoculated with *P. aeruginosa* or *A. baumannii* or *S. aureus* and were subsequently treated twice daily for 3 days, at which time bacterial burden was quantified.

**FIG 3:**
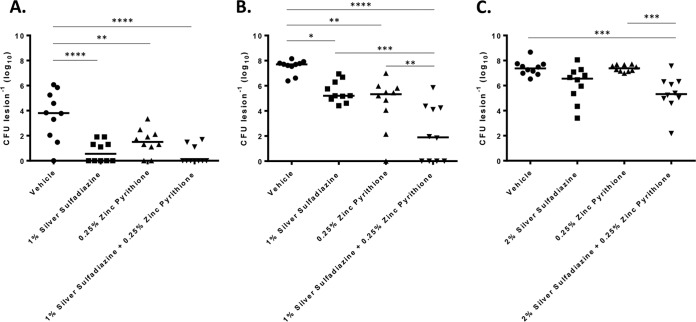
Murine wound decolonization. The number of CFU recovered per lesion (*y* axis) following 3-day treatment with PEG-based ointment containing the indicated agent or mixture (*x* axis) is shown. The results shown are for *S. aureus* (A), *A. baumannii* (B), and *P. aeruginosa* (C). Significant reductions in bacterial burden between treatment groups are indicated (one-way ANOVA [*n =* 10]). *, *P* ≤ 0.05; **, *P* ≤ 0.002; ***, *P* ≤ 0.0005; ****, *P* < 0.0001.

As shown in Fig. 3A, treatment of *A. baumannii*-inoculated wounds with 1% SSD (alone) resulted in an approximately 3-log decrease in bacterial burden (10 CFU per lesion) in comparison to the results seen with animals that were treated with the vehicle alone (9.1 × 10^3^ CFU per lesion). ZnPT treatment resulted in 1.8 × 10^1^ CFU per lesion, with no bacteria recovered from 2 (20%) of the 10 animals within the treatment group. While the improvement was not statistically significant in comparison to the results seen with animals treated with SSD (alone), the combination of 1% SSD and 0.25% ZnPT appeared to display the greatest antimicrobial efficacy. No bacteria were recovered from 7 (70%) of the 10 animals treated, whereas 20 to 90 CFU were recovered from the remaining animals. Tests of *S. aureus*-inoculated wounds revealed that treatment with 1% SSD (alone) resulted in an approximately 2-log decrease in bacterial burden (3.34 × 10^5^ CFU per lesion) in comparison to treatment with vehicle alone. Similarly, treatment with 0.25% ZnPT resulted in a 2-log decrease in *S. aureus* colonization (3.9 × 10^5^ CFU per lesion), with no colony-forming units recovered from 1 (10%) of the 10 animals in the treatment group. The greatest antimicrobial efficacy was observed for the combination-treated group, the members of which displayed an approximately 5.3-log decrease in *S. aureus* burden (9.6 × 10^1^ CFU per lesion) in comparison to those with vehicle-treated wounds, and the combination treatment results showed that no colony-forming units were recoverable from 4 (40%) of the animals within the group (Fig. 3B). Treatment of *P. aeruginosa*-inoculated wounds revealed that, while 0.25% ZnPT (alone) treatment did not result in a significant reduction in bacterial burden in comparison to vehicle treatment (2.5 × 10^7^ CFU versus 2.4 × 10^7^ CFU), 2% SSD (alone) treatment elicited an approximately 1.2-log decrease in *P. aeruginosa* colonization (3.4 × 10^6^ CFU per lesion). The combination of 2% SSD plus 0.25% ZnPT displayed a significant improvement in efficacy, resulting in an approximately 2-log decrease in bacterial burden (2.3 × 10^5^ CFU per lesion; Fig. 3C). Considering that the combination of SSD and ZnPT appeared to display the most antimicrobial efficacy for each pathogen tested, mixtures of the two agents may provide an improved topical agent for the prevention and/or treatment of wound site infections. Accordingly, we next sought to explore this possibility further by evaluating whether the combination ointment negatively affects the normal wound healing process.

### Effects of silver sulfadiazine and zinc pyrithione on wound healing.

Both silver sulfadiazine and zinc pyrithione are FDA-approved topical medications with established, favorable safety profiles. However, their combination has not been evaluated with respect to sterile wound healing. Thus, sterile dermal wounds were created and mice were treated with vehicle or 2% SSD or 0.25% ZnPT or the combination twice daily for 14 days. Each day, animals were assessed for overt signs of wound toxicity, including alertness, grooming, weight, and wound contraction. It should be noted that although 1% silver sulfadiazine is typically used in commercial preparations, concentrations as high as 10% SSD can be used clinically (29); 2% SSD was chosen for evaluation here as it was the concentration required for sufficient activity against *P. aeruginosa*.

**FIG 4:**
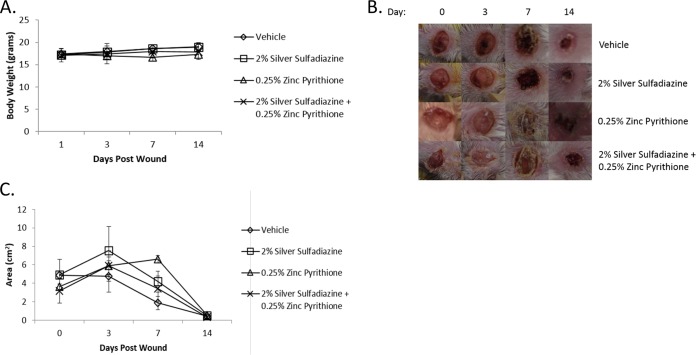
Effects of PEG-based ointments on wound healing and animal health. Panel A shows the average (*n =* 3) body weights of animals (*y* axis) at the indicated days (*x* axis) of treatment with the PEG-based ointments containing the indicated agent or mixture. Panel B shows representative wound healing images following days 0, 3, 7, and 14 of treatment with PEG-based ointments containing 2% silver sulfadiazine or 0.25% zinc pyrithione or their combination. Panel C shows average (*n =* 3) wound contraction corresponding to the images in panel B.

Results revealed no significant difference in levels of body weight for any of the treatment groups (*n =* 3), in comparison to vehicle ointments (Fig. 4A). All mice, regardless of treatment, demonstrated full wound contraction and healing by day 14. However, mice treated with ZnPT alone demonstrated a prolonged initial healing time, with the maximum wound size found at day 7 in contrast to day 3 in all other treatment groups (Fig. 4B and C). Additionally, treatment with ZnPT ointment resulted in fur matting adjacent to the wound (personal observation) which may have been due to licking. Interestingly, the combination ointment treatment did not result in prolonged healing time or cause fur matting. These early toxicity results support the idea of the potential safety of the combination of ZnPT and SSD in an ointment preparation.

## DISCUSSION

The treatment of both acute and chronic wound infections is of central importance to hospitals and health care systems that are actively striving to meet current patient safety benchmarks. The nature and complexity of wound infections are highly dependent on the type of wound as well as on underlying patient comorbidities, although common etiologic organisms identified in most wounds include *S. aureus*, *P. aeruginosa*, *A. baumannii*, *E. coli*, and *Enterococcus* spp. (5, 6, 30). While acute wound infections are often treated successfully with systemic antibiotics, use of a topical drug is frequently warranted, particularly in cases of burn-associated wounds, ischemic wound beds with limited vascular support, chronic colonization, or infection by a multidrug-resistant organism. Nonantibiotic topical medications have the advantages of minimization of systemic side effects, ease of use, direct application to infected tissue, and activity toward otherwise antibiotic-resistant bacteria.

The use of silver sulfadiazine-based products, including ointments, creams, and impregnated dressings, has become a common prophylactic and adjunctive strategy for treatment of many wounds, particularly those that are associated with burns. Silver treatment provides the advantage of broad-spectrum bactericidal activity together with the ability to disrupt biofilms and has been shown to promote wound decolonization (14, 23, 31). However, despite widespread use of silver-based products and supporting *in vitro* and animal model data, there is continued debate regarding clinical infection outcomes in humans, with concerns related to delayed wound healing and emerging resistance (32, 33). This has prompted efforts to improve the antimicrobial performance of silver sulfadiazine by the addition of cerium (34), 0.2% chlorhexidine (35), and hyaluronic acid (36), with varied success. As such, there remains a need for improved topical antimicrobial therapeutics.

In efforts to improve the efficacy of silver sulfadiazine as a topical antimicrobial, our work initially focused on utilizing an adenylate kinase-based reporter of bacterial cell death (24, 27) to identify FDA-approved compounds that exhibit bactericidal activity toward *P. aeruginosa* biofilms. As noted above, the presence of biofilms in chronic wounds has been well established and creates a bacterial physiological state that is tolerant of most antibiotics. Thus, identifying compounds with bactericidal activity toward bacteria within established biofilms has particular relevance. Zinc pyrithione, a compound well known for its antifungal activity in the clinical setting of seborrheic dermatitis and dandruff, was identified in our initial screen and subsequently confirmed to have *in vitro* antimicrobial activity toward planktonic cultures of the members of the ESKAPE pathogen group. Of note, ZnPT demonstrated improved activity compared to SSD against *E. coli*, *S. aureus*, *K. pneumoniae*, *A. baumannii*, *E. faecium*, *E. faecalis*, and *E. cloacae*. Interestingly, SSD was more effective against planktonic *P. aeruginosa* cultures than ZnPT. This finding, though, provided the rationale for evaluating the combination of ZnPT with SSD *in vitro* and *in vivo*.

Our characterization of the antimicrobial effects of ZnPT and SSD in combination focused on *S. aureus*, *P. aeruginosa*, and *A. baumannii* based on their propensity to cause severe wound site infections. In that regard, the combination proved to have an additive effect on all three organisms during planktonic growth and in established biofilms, suggesting that mixtures of ZnPT-SSD may represent a treatment that is more effective than the use of either agent in isolation. Given that the combination suspended in an ointment preparation did not inhibit the activity of the ZnPT and SSD combination *in vitro*, it was then applied to a murine dermal acute wound infection model. The bacteria were completely eradicated from the wound site in 70% of the mice infected with *A. baumannii*, and 40% of the mice infected with *S. aureus* showed eradication of bacteria. Results in *P. aeruginosa* wounds were more modest, with a 2 log reduction in CFU burden following treatment with a ZnPT and SSD combination ointment.

As both ZnPT and SSD are current FDA-approved drugs with favorable safety profiles, significant adverse effects were not anticipated. The rate of wound contraction was not affected by treatment with SSD alone or ZnPT-SSD in combination, although wound contraction was mildly delayed in mice exposed to ZnPT alone. However, by day 14 of exposure, all wounds had closed and showed full reepithelialization, suggesting that there was no overt cytotoxicity observed with the combination treatment. Furthermore, there was no overt cytotoxicity observed with 2% SSD and 0.25% ZPT, alone or in combination.

Taken together, our results support the idea of the use of the combination of ZnPT and SSD as an improved alternative compared to the use of either compound in isolation against common wound-associated organisms. Furthermore, given that both compounds are already FDA-approved topical therapeutics, their combination is particularly attractive for patient safety and expedited drug development. Silver-based products are currently a major component of topical wound therapy, with expansion into impregnated dressings and coatings on medical devices. Given the existing limitations of silver-based therapies, the additive antibacterial effects of SSD and ZnPT may allow more-effective treatment and improved patient outcomes.

## MATERIALS AND METHODS

### Bacterial strains and animals.

The bacterial strains used in these studies included *Staphylococcus aureus* strain UAMS-1, an antibiotic-susceptible clinical isolate that demonstrates robust biofilm formation (37); *Acinetobacter baumannii* strain 98-37-09, which is a well-defined biofilm-forming clinical isolate (38); and the prototypical *Pseudomonas aeruginosa* laboratory PAO1 strain (provided by Barbara Iglewski, University of Rochester, Rochester, NY). Unless otherwise indicated, bacteria were grown overnight in tryptic soy broth (TSB; *S. aureus*) or Luria-Bertani broth (LB; *A. baumannii* and *P. aeruginosa*) and then used to inoculate fresh media (1:100 dilution), grown to early exponential phase (1 × 10^8^ CFU/ml), and processed as described below. Female BALB/c mice 4 to 6 weeks of age were obtained from Charles River Laboratories International, Inc. (Wilmington, MA) and housed according to approved protocol UCAR-2013-024 of the University of Rochester Medical Center, Council on Animal Research (UCAR).

### Chemicals and compound libraries.

A library of 853 Food and Drug Administration (FDA)-approved drugs was obtained from Selleck Chemical (Houston, TX). Zinc pyrithione (ZnPT) was purchased from Sigma-Aldrich (St. Louis, MO). Silver sulfadiazine (SSD) was obtained from TCI America (Portland, OR). ToxiLight BioAssay kits were acquired from Lonza (Basel, Switzerland).

### Adenylate kinase bactericidal reporter assay.

An adenylate kinase (AK) reporter assay of bacterial cell death was used to screen Selleck compound library members for agents that displayed bactericidal activity toward established *P. aeruginosa* biofilms, as previously described (24, 27). As a prerequisite to doing so, the assay’s performance in the high-throughput setting was evaluated by measuring the Z-factor score of mock (DMSO; negative control)- and ciprofloxacin (20 µg·ml^−1^; positive control)-treated *P. aeruginosa* biofilm cells in 96-well plate format. To do so, overnight cultures of *P. aeruginosa* strain PAO1 were used to seed individual wells of a flat-bottom plate (Falcon; Corning Life Sciences, Durham, NC) containing 200 µl of fresh LB medium (~1 × 10^7^ CFU ml^−1^ [final concentration]). Plates were incubated at 37°C for 72 h to allow biofilm formation. Nonadherent cells were aspirated, biofilms were washed twice with phosphate-buffered saline (PBS), and alternating columns were treated with fresh media containing 2 µl of either dimethyl sulfoxide (DMSO) or ciprofloxacin. Plates were incubated for 24 h at 37°C and then processed to compare the effects of antibiotic treatment on biofilm-associated bacteria by measuring adenylate kinase release into the biofilm supernatant and determining the number of bacteria remaining. More specifically, biofilm supernatants were transferred to 96-well white-walled flat-bottomed plates (Corning, Inc.) for adenylate kinase detection, as described below, whereas the numbers of biofilm-associated bacteria remaining under DMSO and ciprofloxacin treatment conditions were determined by resuspending the biofilm in 100 µl LB and plating for colony-forming units. The amount of adenylate kinase released into the supernatant was measured in the dark by adding AK detection reagent (100 µl) for 30 min at room temperature and analyzing levels of luminescence at all wavelengths (from 250 nm to 850 nm) with an integration time of 1,000 ms per well on a SpectraMax M5 plate reader. A comparison of the levels of AK-based luminescence of DMSO- and ciprofloxacin-treated biofilms revealed a Z′-factor score of 0.90 ± 0.11, which corresponded to a ≥9-log reduction in the burden of *P. aeruginosa* biofilm-associated cells, indicating that the assay is amenable to identifying agents that display bactericidal activity toward *P. aeruginosa* biofilms (39). For Selleck library screening, 72-h *P. aeruginosa* PAO1 biofilms were grown and processed as described above, except that biofilms were treated with 50 µM of individual members of the library; the first position and last position of each plate were treated with 20 µg·ml^−1^ ciprofloxacin (positive control) or DMSO (1% final concentration; negative control).

### Antimicrobial susceptibility testing.

MIC testing was performed according to Clinical and Laboratory Standards Institute guidelines (40). Briefly, 1 × 10^5^ CFU of the indicated bacterial strain was added to individual wells of a microtiter plate containing 88 µl of Mueller-Hinton broth (MHB) media and 2-fold-increasing concentrations of test agent (0 to 128 µg·ml^−1^). Plates were incubated for 16 h at 37°C, and the lowest concentration of the agent that inhibited *P. aeruginosa* growth was considered to be the MIC. Fractional inhibitory concentration (FIC) testing index calculations were performed to measure interactions between SSD and ZnPT in 96-well microtiter plates, as previously described (41). Briefly, in checkerboard format, each row of the plate contained increasing concentrations of SSD (in 2-fold increments; 0 to 128 µg·ml^−1^), whereas each column contained increasing concentrations of ZnPT (in 2-fold increments; 0 to 128 µg·ml^−1^). The FIC was determined using the following formula: (MIC of drug A in combination/MIC of drug A alone) − (MIC of drug B in combination/MIC of drug B alone) = FIC. A synergistic interaction was defined as an FIC value of ≤0.5, an additive interaction as an FIC of 0.5 to 1.0, no interaction as an FIC of 1 to 4, and an antagonistic interaction as an FIC of >4 (41).

To measure the antimicrobial effects of the test agents on established *S. aureus*, *A. baumannii*, and *P. aeruginosa* biofilms, 24-h to 72-h biofilms were created as previously described for *S. aureus* (42) and *A. baumannii* (43) or as described above for *P. aeruginosa*. Biofilms were washed to remove nonadherent cells, and 100 µl of fresh media was added to each well such that the wells contained increasing concentrations of SSD (in 2-fold increments; 0 to 128 µg·ml^−1^), ZnPT (2-fold increments; 0 to 128 µg·ml^−1^), or the combination (2-fold increments; 0 to 128 µg·ml^−1^). Plates were incubated for 24 h, at which point the supernatant was removed, the biofilm was resuspended in 0.8% NaCl, and the biofilm-associated cells were enumerated by plating on MH agar (Fisher Scientific).

### Preparation of test article ointments.

The polyethylene glycol (PEG) ointment base was prepared by mixing PEG 400 (70% [wt/vol]) with PEG 3350 (30% [wt/vol]) as described by the United States Pharmacopeia and the National Formulary (44). SSD and ZnPT were suspended in 250 µl of DMSO to create working concentrations of 100 mg and 12.5 mg, respectively. Mixtures were then added directly to 5 g of PEG ointment that had been preliquefied by heating at 60°C for 10 min to create 2% SSD–0.25% ZnPT suspensions and then cooled to room temperature to solidify the suspension. The same procedure was used to create DMSO vehicle control and 1% SSD–0.25% ZPT PEG mixtures by adding a combination of 50 mg SSD and 12.5 mg ZPT in a total of 250 µl DMSO.

### *In vitro* ointment antimicrobial testing.

Antimicrobial zones of inhibition were measured for PEG ointment compilations using the indicated bacterial strains, as previously described (28). To do so, 100 µl of 1 × 10^8^ CFU ml^−1^ of *A. baumannii*, *S. aureus*, or *P. aeruginosa* was spread on MH plates using a sterile glass hockey stick. Plates were dried for 10 min, and 40 µl of the indicated ointment was added to the center of the plate. Plates were incubated at 37°C for 16 h, and zones of bacterial clearance were measured using ImageJ software (National Institutes of Health, Bethesda, MD).

### Dermal wound model of infection and treatment of mice.

SSD and ZnPT ointment preparations were evaluated for *in vivo* antimicrobial activity toward *A. baumannii*, *S. aureus*, or *P. aeruginosa* using a dermal wound model (45), with modifications. Mice were anesthetized by intraperitoneal injection with a mixture of 100 mg·ml^−1^ ketamine (Hospira Inc., Lake Forest, IL) and 20 mg·ml^−1^ xylazine (Lloyd Laboratories, Shenandoah, IA) with 0.9% NaCl at 5 µl per 1-g body weight. Pain relief in the form of 20 µl 0.5% bupivacaine (Sensorcaine; APP Pharmaceuticals, Schaumburg, IL) was administered prior to dermal wounding. The dorsal midsection of the mouse was shaved and cleaned with a series of Betadine scrubs (Fisher Scientific), povidone-iodine pads (Professional Disposables International Inc., Orangeburg, NY), and isopropyl alcohol pads (Fisher Scientific) for a total contact time of 2 min. A single wound was created in this sterile field on the mouse with a 6-mm-diameter biopsy punch (Fisher Scientific) to remove only the dermal layer and not disrupt the underlying musculature. The wounds of the mice were inoculated with 1 × 10^7^ of the indicated bacterial strain by pipetting 10 µl of culture directly onto the wound. Mice were then treated with ointment formulations (50 µl) containing vehicle alone (5% DMSO) or 1% SSD or 2% SSD or 0.25% ZnPT or 1% SSD–0.25% ZnPT or 2% SSD–0.25% ZnPT at 45 min postinoculation; treatments were repeated every 12 h for 3 days. Mice were then euthanized via CO_2_ asphyxiation and cervical dislocation, and the wound and underlying muscle were excised with an 8-mm-diameter biopsy punch and placed in microcentrifuge tubes containing 1 ml of freshly made PBS. Samples were homogenized for 5 min and plated, and the bacteria were enumerated.

### *In vivo* toxicity testing.

Ointment toxicity was tested in a modified dermal wound model. Mice in groups of three per indicated treatment group were wounded as described above but were not inoculated with bacteria. The wound was treated with vehicle or 2% SSD or 0.25% ZnPT or 2% SSD–0.25% ZnPT combination ointment twice daily for 14 days. Mice were weighed and assessed for grooming and alertness, and images of the wound were obtained daily to measure wound contraction using ImageJ (NIH). Wound contraction was calculated as the percentage of wound area reduction using the following formula: WC*_d_* = (1 − WA*_d_*/WA_0_) × 100, where WC is wound contraction, WA is wound area, *d* is day, and 0 indicates initial day, as previously described (46).

### Statistical analyses.

Analyses were performed using GraphPad Prism software version 6.0 (GraphPad Software, Inc., La Jolla, CA). For zone-of-inhibition assays, a Student’s *t* test was used to determine the statistical power of the comparisons between treatment groups. For murine studies, measures were log transformed and subjected to a one-way analysis of variance (ANOVA) to determine the statistical power.
